# Correction to ‘ChEC-seq2: an improved chromatin endogenous cleavage sequencing method and bioinformatic analysis pipeline for mapping in vivo protein–DNA interactions’

**DOI:** 10.1093/nargab/lqaf059

**Published:** 2025-05-13

This is a correction to: Jake VanBelzen, Chengzhe Duan, Donna Garvey Brickner, Jason H Brickner, ChEC-seq2: an improved chromatin endogenous cleavage sequencing method and bioinformatic analysis pipeline for mapping in vivo protein–DNA interactions, NAR Genomics and Bioinformatics, Volume 6, Issue 1, March 2024, lqae012, https://doi.org/10.1093/nargab/lqae012

The supplementary step-by-step protocol omitted an ingredient (2% SDS) from the 2x Stop Solution.

The corrected supplementary file is published with this correction notice.

This correction does not affect the results, discussion and conclusions presented in the article. These details have been corrected only in this correction notice to preserve the published version of record.

## Supplementary data

Supplementary data is available at NAR Genomics & Bioinformatics online.

## Supplementary Material

lqaf059_Supplemental_File

