# Supplementary material for: ChEC-seq2: an improved chromatin endogenous cleavage sequencing method and bioinformatic analysis pipeline for mapping in vivo protein–DNA interactions
Source: NAR Genom Bioinform. 2024 Feb 7;6(1):lqae012. doi: 10.1093/nargab/lqae012 (PMC10849192; doi:10.1093/nargab/lqae012)
Supplement: lqae012_Supplemental_Files [file lqae012_supplemental_files.zip › Supplementary Materials.docx]

**Supplementary Materials**

**Figure legends**

**Figure S1. Consensus motifs for Rap1, Gcn4 and Ino2.** Motifs for Rap1, Gcn4 and Ino2 from *Saccharomyces cerevisiae* from https://jaspar.elixir.no/.

**Figure S2. TapeStation size analysis of ChEC samples.** TapeStation traces of molecular weight standards (top) or uncleaved genomic DNA isolated from two different strains (**a**), DNA from three replicates from Rap1-MNase after 2 minutes of cleavage **(b)**, DNA from three replicates of soluble MNase after 15 seconds of cleavage **(c),** DNA treated with three different Tn5 concentrations **(d)** and final amplified libraries from three different replicates of Rap1-MN ChEC **(e)**. Note: * indicates the internal size standards.

**Figure S3. Effect of trimming reads to the first base pair on peak shape.** Untrimmed (top) or trimmed (bottom; only the first base was tallied) reads from Rap1-MN ChEC-seq2 were plotted over the *PGK1* gene. Genes on the top strand are transcribed left to right and genes on the bottom strand are transcribed right to left. The light grey box indicates a dubious open reading frame (*YCR013C*).

**Figure S4. Filtering ChEC peaks to identify high-confidence sites. (a)** Flowchart for DoubleChEC. **(b)** The distribution of nearest distances between local maxima identified from Rap1-MN, Gcn4-MN and Ino2-MN cleavage sites. **(c)**  The number of initial local maxima, sites that were significantly enriched over sMNase and doublets identified from Rap1-MN, Gcn4-MN and Ino2-MN cleavage sites. **(d)** Top scoring motifs identified by MEME analysis of peaks (± 50 base pairs) identified as significantly enriched over soluble MNase.

**Figure S5. Comparison of different chromatin accessibility assays.** (a) Mean cleavage frequency for Prp20 or sMNase from cells grown in SDC-histidine across the repressed *GAL1-10* locus. (b) Mean cleavage frequency for Htz1 or sMNase from cells grown in SDC-inositol across the repressed *GAL1-10* locus. (c) Meta-promoter analysis for Prp20 and sMNase from SDC-histidine (left) or Htz1 and sMNase from SDC-inositol (right). Mean CPM normalized cleavage from 700 base pairs upstream to 200 base pairs downstream of the transcriptional start site of 5348 genes with mapped transcriptional start sites (<http://yeastss.org/> ; 35). (d) MEME analysis of high-confidence peaks identified for Gcn4 using Prp20 as a negative control (top) or Ino2 using Htz1 as a negative control (bottom).

**Figure S6. Inducible TFs show increased cleavage over their high-confidence sites.** Mean cleavage by Gcn4 **(a)** or Ino2 **(b)** over high-confidence sites from cells grown in media ± histidine **(a)** or ± inositol **(b)**.


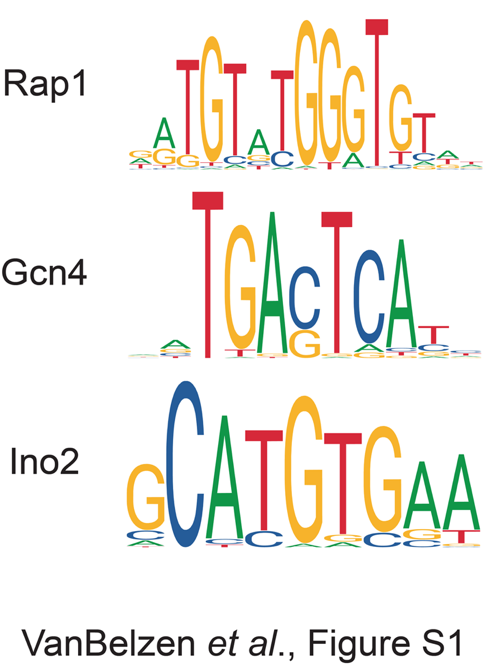


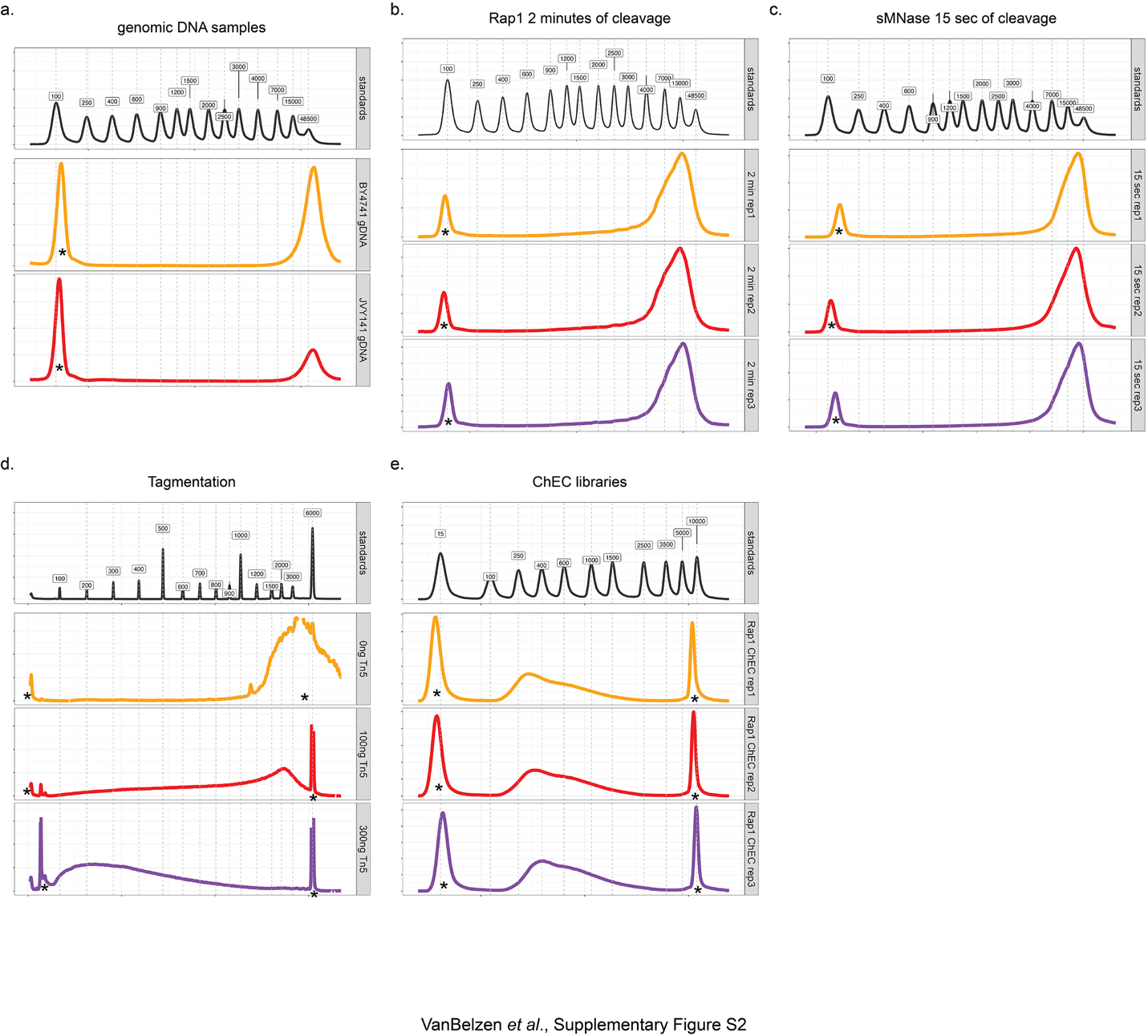


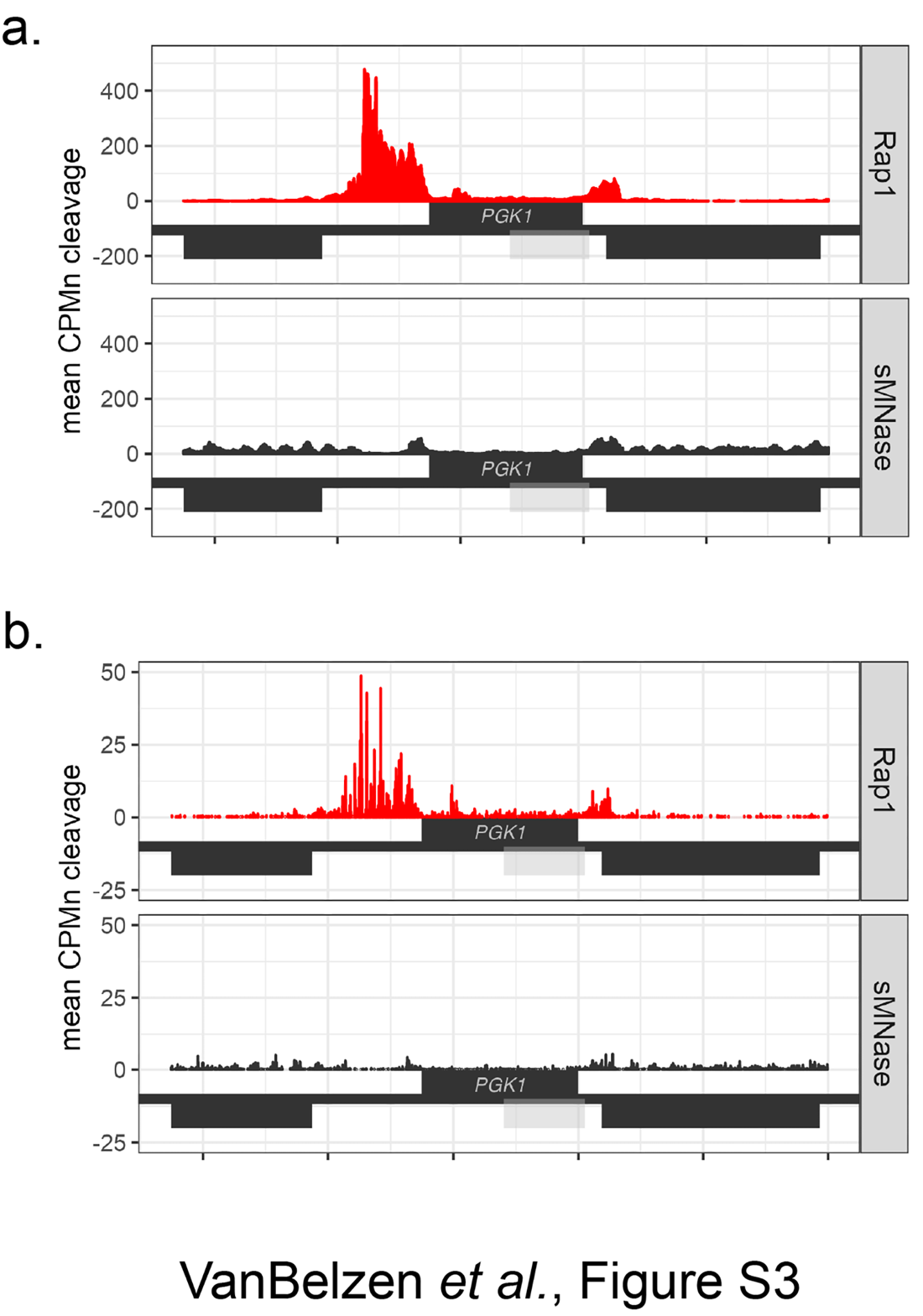


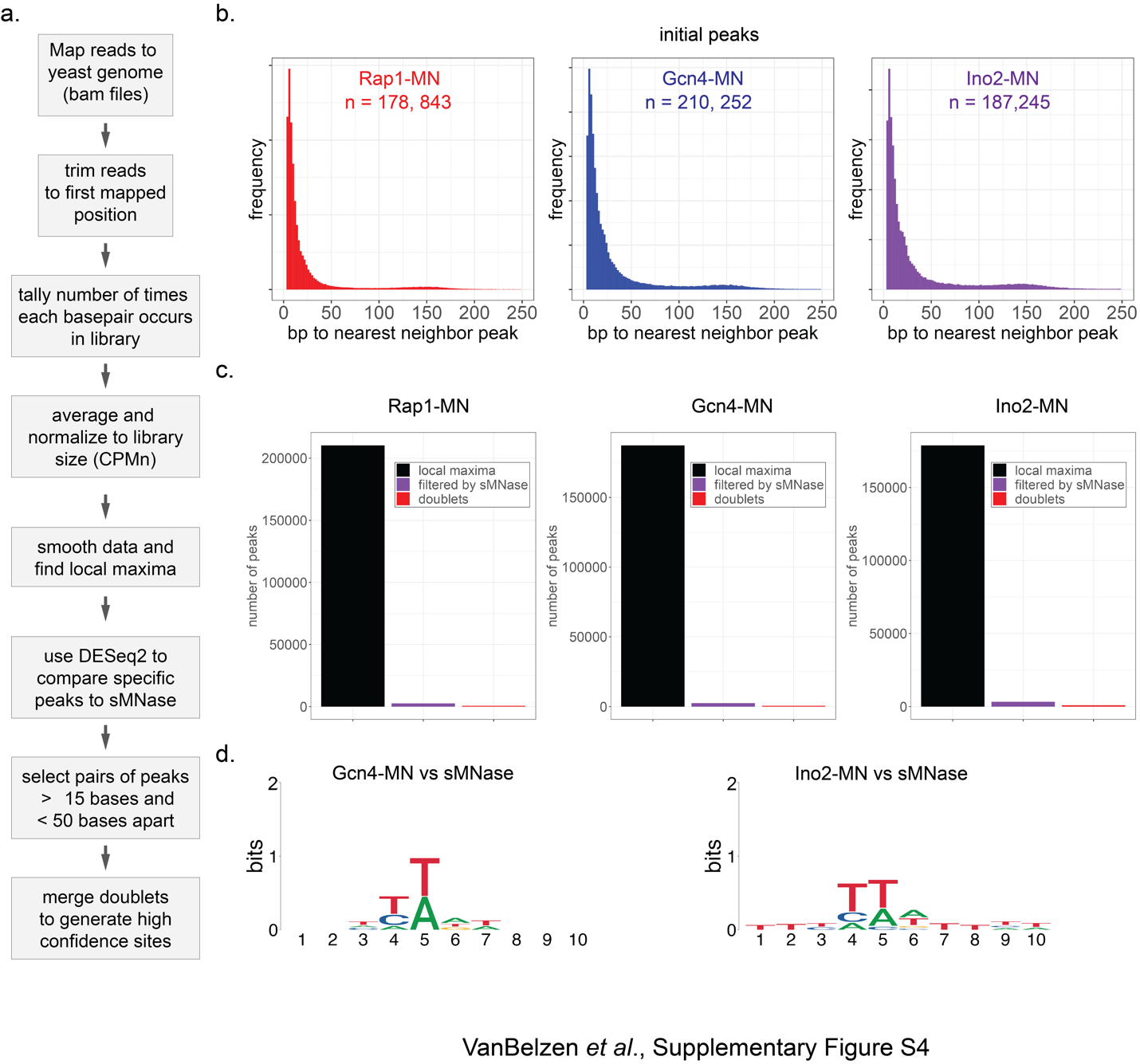


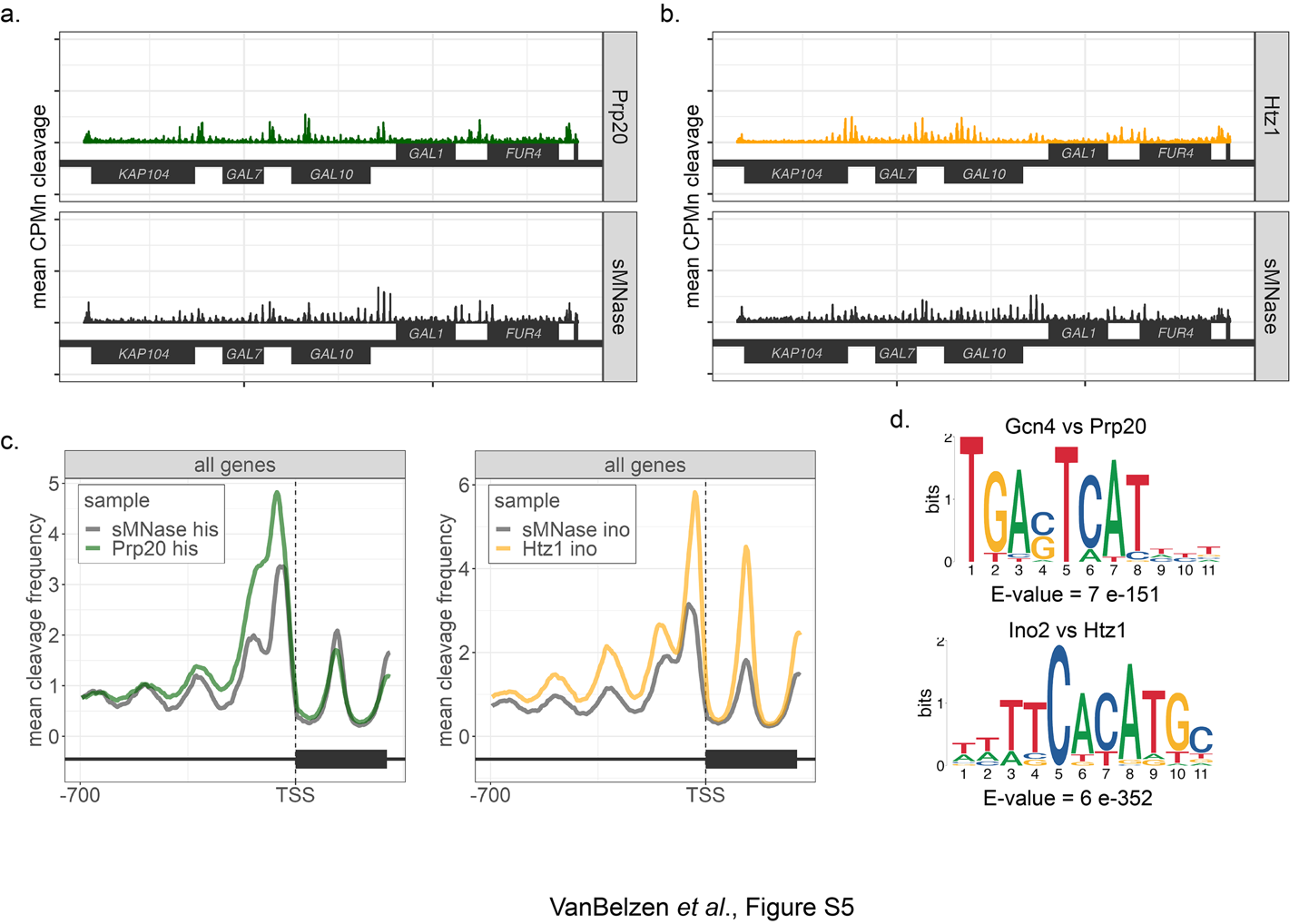


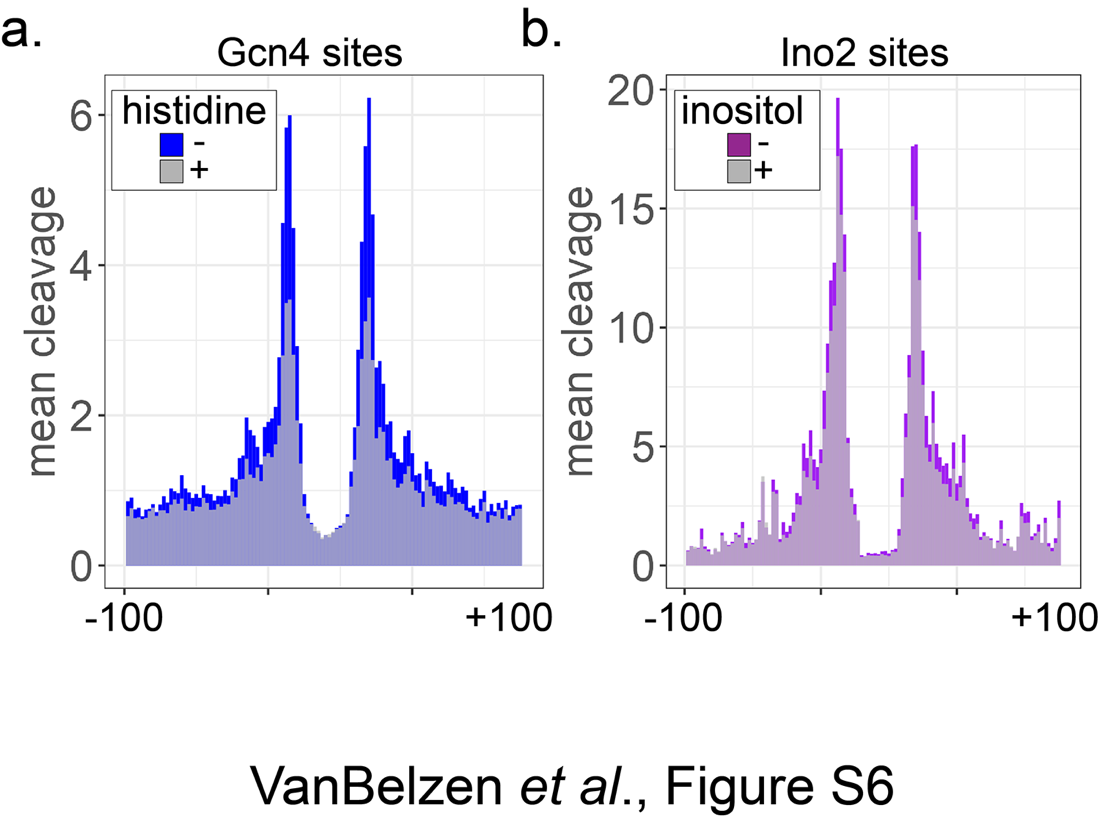


**Supplementary Table S2**

| **Strain** | **Genotype** |
| --- | --- |
| DBY2222 | *his3∆1 leu2∆1 met15∆0 ura3∆0 Prp20-Mnase:KanMx* |
| DBY2238 | *his3∆1 leu2∆1 met15∆0 ura3∆0 Gcn4-Mnase:KanMx* |
| DBY2309 | *his3∆1 leu2∆1 met15∆0 ura3∆0 Ino2-Mnase:KanMx* |
| DBY2310 | *his3∆1 leu2∆1 met15∆0 ura3∆0 Htz1-Mnase:KanMx* |
| JVY141 | *ura3∆0 leu2∆0 met15∆0 HIS3:sMNase* |
| JVY207 | *his3∆1 leu2∆1 met15∆0 ura3∆0 Rap1-Mnase:KanMx* |

**Chromatin endogenous cleavage protocol**

**Chromatin digestion**

1. Grow cells in 10ml overnight at 30°C, 200 rpm.
2. Dilute cultures into 50ml media to OD_600_ ~ 0.1.
3. When cultures reach OD_600_ = 0.5 - 0.8, harvest 25 ODs (*i.e.* 50ml if the OD600 = 0.5) of cells by centrifugation at 2500 x *g* for 1 minute.
4. Resuspend cells in 1 ml Buffer A and transfer to a 1.5 ml tube.
5. Pellet cells by centrifugation at 2500 x *g* for 1 minute, remove supernatant.
6. Wash cells 2 x 1 ml Buffer A, removing supernatant.
7. Resuspend cells in 600 µl Buffer A + 0.1% Digitonin.
8. Transfer tube to a 30°C heat block and incubate for 5 minutes.
9. Add 5 µl of 333 mM CaCl_2_, mix by inverting, incubate at 30°C for the appropriate cleavge time (determined empirically for each protein).
10. To stop the reaction, remove 200 µl cells and combine with 200 µl 2x Stop Buffer.
11. Add 8 µl Proteinase K (20 µg/µl) and mix.
12. Incubate at 50°C, agitating 800 rpm for 30 minutes in a thermomixer.
13. Remove samples from thermomixer and cool at room temperature for 5 minutes.
14. Add 400 µl Phenol-Chloroform-Isoamyl Alcohol (25:24:1), pH 7.8, mix.
15. Centrifuge at 24,000 x *g*, 5 minutes.
16. Transfer aqueous phase to a phase-lock tube.
17. Add 200 µl Phenol-Chloroform-Isoamyl Alcohol (25:24:1), pH 7.8.
18. Invert 10x to mix.
19. Centrifuge at 24,000 x *g* for 5 minutes.
20. Transfer aqueous phase to a tube containing 1 ml 100% Ethanol.
21. Add 2 µl of linear acrylamide (5 µg/µl).
22. Invert 10x to mix.
23. Incubate at -80°C for 30 minutes.
24. Centrifuge at 24,000 x g, 4°C for 10 minutes.
25. Pour off supernatant.
26. Wash DNA pellet in 1 ml of 70% ethanol.
27. Centrifuge at 24,000 x g for 1 minute.
28. Pour off supernatant. Collect residual ethanol by centrifugation and remove by pipetting.
29. Dry DNA pellet until all ethanol had evaporated.
30. Add 58 µl of 10 mM Tris-HCl, pH 8.5 to DNA pellet.
31. Incubate overnight at room temperature.
32. Incubate at 37°C for 30 minutes.
33. Add 2 µl RNase A (10 µg/µl) to DNA.
34. Incubate at 37°C for 30 minutes.
35. Evaluate molecular weight of DNA by gel electrophoresis, 0.8% agarose or TapeStation.
36. Quantify DNA concentration with the Qubit double-stranded DNA, Broad Range Assay.
37. Stored DNA at 4°C until library preparation was performed (up to a month), then stored at -20°C.

**Buffer A**

15 mM Tris-HCl, pH 7.5

80 mM KCl

0.1 mM EGTA

1.0 mM PMSF

0.5 mM Spermidine

0.2 mM Spermine

-Add 1 EDTA-Free Protease Inhibitor Tab per 50 ml Buffer A (Roche; Sigma # 11873580001)

**2x Stop Buffer**

400 mM NaCl

20 mM EDTA

4 mM EGTA

**Library Preparation & Sequencing**

**End repair and ligation**

1. Dilute each DNA sample to 10 ng/µl x 30 µl in PCR tubes.
2. For each sample, in a 0.2ml PCR tube, combine:

2.5 µl 10x CutSmart Buffer

1.5 µl water

20µl cleaved genomic DNA (10ng/µl)

1µl Quick CIP (NEB #M0508)

1. Mix carefully and then spun tubes to collect droplets.
2. Heat in a thermocycler:
   - 37°C - 10:00
   - 80°C - 5:00
   - 4°C - HOLD
3. Extend DNA ends and add a 5’ phosphate, add 10µl of the following to each reaction:
   - 1 µl 10x CutSmart Buffer (NEB #E1201)
   - 3.5 µl 1 mM dNTPs (NEB #E1201)
   - 4.5 µl water
   - 1µl Blunting Enzyme Mix (NEB #E1201)
4. Heat in a thermocycler:
   - 25°C - 30:00
   - 70°C - 10:00
   - 4°C - HOLD
5. Ligate i5 Y-Adapter to cleaved, repaired gDNA (NEB #M2200). To each reaction, add:
   - 32.5 µl 2x Quick Ligation Buffer (NEB #M2200)
   - 0.5 µl 45 µM i5 Y-Adapter (NEB #M2200)
   - 2 µl Quick Ligase (NEB #M2200)
   - 32.0 µl water
   - Total Volume: 100 µl
6. Heated in a thermocycler:
   - 25°C - 20:00
   - 4°C - HOLD
7. Allow ligation reactions to come to room temperature.
8. Add 70 µl SPRIselect beads (Final [Beads] = 0.7x) to each 100 µl ligation reaction (PCR strip tubes) and mix by pipetting 20x.
9. Incubate at room temperature for 1 minute,
10. Place PCR tubes onto magnetic stand and waited for beads to adhere, carefully remove supernatant.
11. Add 180 µl of 85% ethanol, waited 30 seconds, and then remove ethanol.
12. Repeat ethanol wash and carefully remove any residual ethanol.
13. Air-dry beads for 3 minutes.
14. Remove tube from rack and resuspend beads in 25 µl of water.
15. Incubate at room temperature for 1 minute, then place tube back onto magnetic stand and wait for beads to adhere.
16. Collect DNA-containing supernatant and transfer to new PCR tubes.
17. Quantify DNA concentration with 2 µl using Qubit 1x High Sensitivity dsDNA kit.
18. Store purified, ligated DNA at 4°C overnight.

**Tagmentation and library amplification**

1. To 10 ng of ligated ChEC-gDNA in a 24 µl reaction volume, add:
   - 6 µl 4x Tagmentation Buffer (see below)
   - 6 µl 100% DMF (dimethylformamide)
   - ~ µl 10 ng DNA
   - 1 µl Tn5 (300ng), loaded with i7-A
   - Water to 24 µl
2. Mix reactions by flicking wells, and then spin plate to collect droplets.
3. In a thermocycler, heat at 55°C for 10 minutes, then hold at 10°C
4. Add 16.8 µl SPRIselect beads to each 24 µl Tagmentation reaction (Final [Beads] = 0.7x) in a PCR tube.
5. Mix by pipetting 20x.
6. Incubate at room temperature for 1 minute
7. Place tubes onto magnetic stand and wait for beads to adhere
8. Carefully remove supernatant
9. Add 180 µl of 85% ethanol, wait 30 seconds, and then removed ethanol.
10. Repeat ethanol wash and remove any residual ethanol.
11. Air-dry beads for 3 minutes
12. Remove tubes from rack and resuspend beads in 10 µl of water.
13. Incubate at room temperature for 1 minute, then place tube back onto magnetic stand and wait for beads to adhere.
14. Collect supernatant and transfer to new PCR tubes.
15. Prepare library amplification reaction:
    - 6 µl 2x KAPA Library Amplification Master Mix
    - 0.5 µl 10 µM S502 Index-Primer ([Final] = 0.42 µM)
    - 0.5 µl 10 µM N7xx Index-Primer ([Final] = 0.42 µM)
    - 5 µl Purified, Tagmented DNA
    - Total Volume = 12 µl
16. Mix reactions and spin to collect droplets.
17. Transfer tubes to a thermocycler and run the following protocol:
    - 72°C - 3:00
    - 98°C - 2:45
    - 98°C - 0:15*
    - 62°C - 0:30* *15 cycles
    - 72°C - 1:30*
    - 72°C - 3:00
    - 4°C - HOLD
18. Add 8.4 µl of SPRIselect beads directly to PCR tubes containing 12 µl of Library Amplification Reaction (Final [Beads] = 0.7x).
19. Mix by pipetting 20x.
20. Incubate at room temperature for 1 minutes
21. Place PCR strip tubes onto magnetic stand and wait for beads to adhere
22. Carefully remove supernatant
23. Add 180 µl of 85% ethanol, wait 30 seconds, and then remove ethanol.
24. Repeat ethanol wash and remove any residual ethanol.
25. Air-dry beads for 3 minutes
26. Remove tubes from rack and resuspend beads in 10 µl of water.
27. Incubate at room temperature for 1 minute, then place tube back onto magnetic stand and wait for beads to adhere.
28. Collect DNA-containing supernatant and transfer to new PCR tubes.
29. Quantify DNA concentration using the QuBit dsDNA High Sensitivity Assay (see table)
30. Combine 2 µl sample with 190 µl Buffer to evaluate molecular weight of libraries on the TapeStation with the D5000 kit.
31. Pool sample libraries with different indexes
32. Combine equimolar amounts of each library into a 1.5 ml tube.
33. The pooled library concentration should be 2 nM in 100µl.

**4x Tagmentation Buffer**

40 mM Tris-HCl, pH 7.5

40 mM MgCl_2_
